# A Putative XIST–miRNA–ZNF662 ceRNA Axis with Diagnostic and Prognostic Potential in Oral Squamous Cell Carcinoma

**DOI:** 10.3390/ijms27093987

**Published:** 2026-04-29

**Authors:** Nowsheen Bhat, Vasileios Panagiotis Lenis, Sahar Mohsin

**Affiliations:** 1 Department of Anatomy, College of Medicine and Health Sciences, United Arab Emirates University, Al Ain 15551, Abu Dhabi Emirate, United Arab Emirates; 2 Department of Cancer and Genomic Sciences, University of Birmingham, Edgbaston, Birmingham B15 2SY, UK

**Keywords:** oral squamous cell carcinoma, head and neck squamous cell carcinoma, ceRNA network, long non-coding RNA, microRNA, ZNF662, XIST, diagnostic biomarker, prognostic biomarker

## Abstract

Oral squamous cell carcinoma (OSCC) remains a major cause of cancer-related morbidity and mortality, and reliable biomarkers for early diagnosis and risk stratification are still lacking. Long non-coding RNAs (lncRNAs) and microRNAs (miRNAs) can regulate gene expression through competing endogenous RNA (ceRNA) interactions, but OSCC-specific ceRNA axes with clinical relevance are still poorly defined. We integrated lncRNA, miRNA, and mRNA expression data from six OSCC-related datasets in the Gene Expression Omnibus with in silico interaction predictions to construct an OSCC-focused ceRNA network and examine its association with survival. The resulting network comprised 8 mRNAs, 22 miRNAs, and 12 lncRNAs. Within this network, we identified a previously unrecognized XIST–miRNA–ZNF662 axis that has not been characterized in OSCC. ZNF662 was consistently downregulated in tumors, and higher ZNF662 expression was associated with improved survival in an independent head and neck squamous cell carcinoma cohort. Components of the XIST–miRNA–ZNF662 axis also showed excellent diagnostic performance for distinguishing OSCC from normal samples across (Gene Expression Omnibus) GEO datasets, highlighting a ceRNA module with promising diagnostic and prognostic potential that could be explored further in non-invasive biofluids.

## 1. Introduction

Oral malignancies, including cancers of the lip and oral cavity, rank among the most common cancers worldwide and are a major cause of cancer-related morbidity and mortality [1,2,3]. According to GLOBOCAN 2022, cancers of the lip and oral cavity account for an estimated 389,846 new cases and 188,438 deaths worldwide, ranking 16th for incidence and 15th for mortality globally [2]. Oral squamous cell carcinoma (OSCC) accounts for most oral malignancies and often presents at advanced stages, when the treatment options are more aggressive and long-term survival is poor. This burden is especially pronounced in regions with high tobacco and betel-quid consumption, where OSCC disproportionately affects men and individuals of lower socioeconomic status [1,2,3].

Major risk factors for OSCC include tobacco (smoked and smokeless), alcohol, betel-quid and areca nut chewing, poor oral hygiene, and chronic mucosal irritation [4,5]. Despite improvements in surgery, radiotherapy, and systemic therapies, 5-year survival for OSCC has remained largely unchanged over recent decades, particularly for patients diagnosed at late stages [4,5]. Conventional clinicopathological parameters (tumor stage, nodal status, histological grade, and margin status) are useful but imperfect tools for risk stratification, and there is a pressing need for molecular biomarkers that can support earlier detection, refine prognosis, and guide personalized management [6].

High-throughput transcriptomic studies have revealed that only a small fraction of the human genome encodes protein-coding genes, whereas the majority is transcribed into non-coding RNAs, including microRNAs (miRNAs) and long non-coding RNAs (lncRNAs) [7,8,9]. Both classes have been implicated in OSCC pathogenesis, influencing proliferation, apoptosis, epithelial–mesenchymal transition (EMT), invasion, and treatment resistance through their effects on gene regulatory networks [10,11,12]. The competing endogenous RNA (ceRNA) hypothesis proposes that lncRNAs, mRNAs, and other transcripts can regulate one another by competing for shared miRNAs via microRNA response elements, thereby forming ceRNA networks with the potential to fine-tune oncogenic and tumor-suppressive pathways [12,13]. Although several individual lncRNAs, miRNAs, and mRNAs have been linked to OSCC, ceRNA axes with integrated evidence for expression, diagnostic, and survival analyses remain poorly defined, and many reported networks rely on single datasets or limited in silico prediction with minimal validation [14,15,16,17,18,19]. X-inactive specific transcript (XIST) has been implicated in oral cancer biology, and ZNF662 has been suggested as a potential tumor suppressor in other malignancies, but their roles in OSCC and their possible integration within ceRNA networks have not been systematically explored. Moreover, previously reported XIST-related ceRNA networks in OSCC have largely focused on individual axes identified within single cohorts or specific experimental settings, rather than adopting a multi-dataset framework, as in our study, that integrates expression, diagnostic, and survival evidence.

Therefore, in this study we integrated multiple Gene Expression Omnibus (GEO) OSCC datasets spanning lncRNA, miRNA, and mRNA expressions to construct an OSCC-focused ceRNA network, intersecting differential expression with three independent target-prediction resources. We then prioritized non-coding RNA centered axes by jointly evaluating tumor versus normal expression, diagnostic performance, and prognostic associations using The Cancer Genome Atlas head and neck squamous cell carcinoma (TCGA-HNSC) cohort. Using this approach, we specifically investigated whether XIST and ZNF662-centered ceRNA axes contribute to OSCC and may have diagnostic and prognostic relevance, providing, to our knowledge, the first integrated expression, diagnostic, and survival-based characterization of an XIST–miRNA–ZNF662 module in this disease. This framework aims to identify ceRNA axes that show coordinated dysregulation, have potential clinical utility, and are linked to key biological processes, thereby providing a hypothesis-generating basis for future mechanistic and clinical studies in OSCC.

## 2. Results

### 2.1. Differential Expression Across Modalities

Differential expression analysis across one miRNA dataset, one lncRNA RNA-seq dataset, and four mRNA datasets identified 56 differentially expressed miRNAs (DEmiRNAs), 2910 differentially expressed lncRNAs (DElncRNAs), and 680, 3905, 3412, and 3866 differentially expressed mRNAs (DEmRNAs) in the four mRNA datasets GSE150469, GSE246050, GSE74530, and GSE30784, respectively (Table 1).

**Figure 1:**
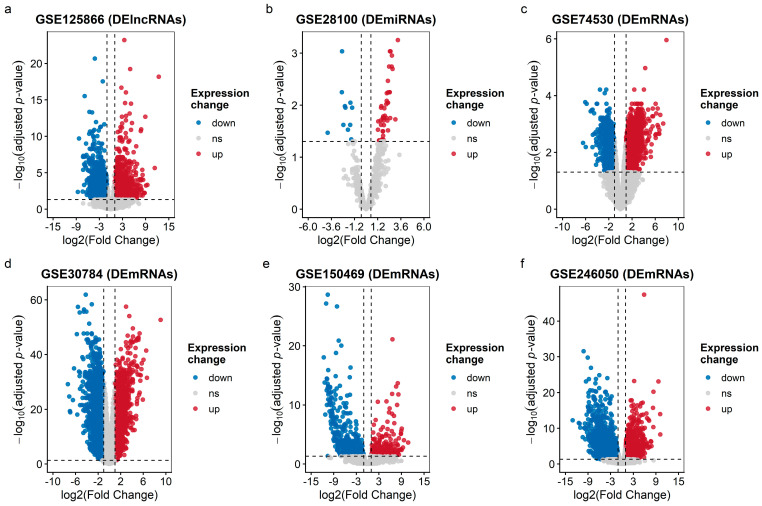
Volcano plots showing differentially expressed RNAs (DE RNAs) across GEO datasets. (**a**) DElncRNAs in GSE125866; (**b**) DEmiRNAs in GSE28100; DEmiRNAs in (**c**) GSE74530 (microarray); (**d**) GSE30784 (microarray); (**e**) GSE150469 (RNA-seq); and (**f**) GSE246050 (RNA-seq). Up-regulated genes are shown in red, down-regulated in blue, and non-significant genes in gray. Differential expression thresholds were |log2FC| > 1 and FDR < 0.05.

**Figure 2:**
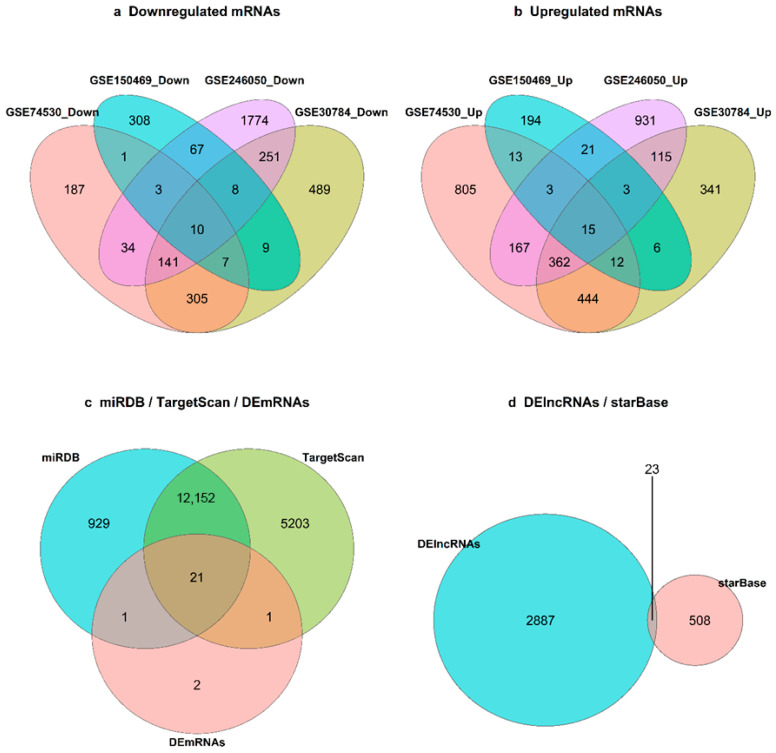
Venn diagrams showing overlaps of differentially expressed genes and predicted targets. (**a**) Overlap of downregulated DEmRNAs across the four mRNA datasets (GSE74530 and GSE30784, microarray; GSE150469 and GSE246050, RNA-seq). (**b**) Overlap of upregulated DEmRNAs across the same four datasets. (**c**) Overlap of predicted mRNA targets for the 56 DEmiRNAs between miRDB and TargetScan and their intersection with the 25 consensus DEmRNAs, yielding 21 common targets. (**d**) Overlap of lncRNA targets predicted by starBase (ENCORI) and DElncRNAs identified in GSE125866, highlighting 22 intersecting lncRNAs.

**Figure 3:**
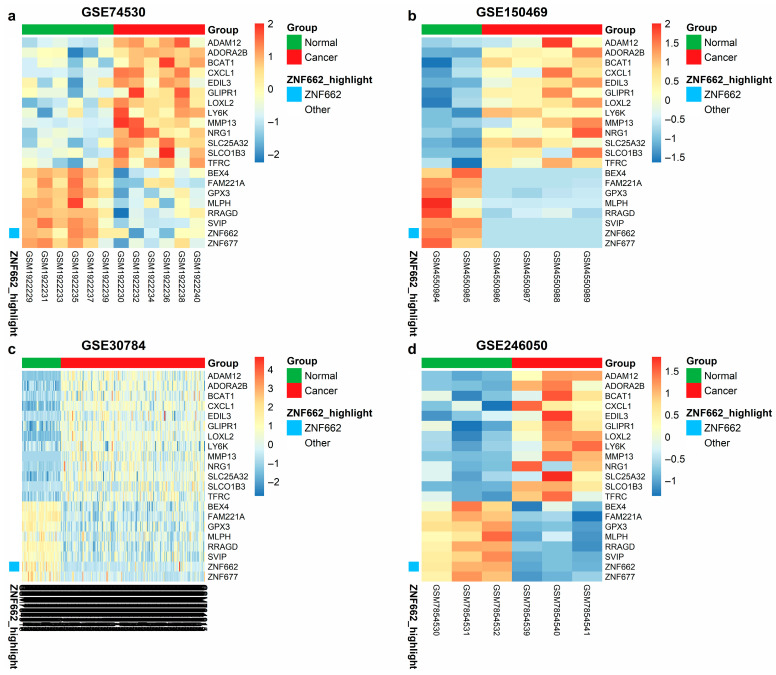
Heatmaps of high-confidence intersecting DEmRNAs across GEO mRNA datasets. The 21 high-confidence consensus DEmRNAs (common across mRNA datasets and supported as miRNA targets) are shown in (**a**,**c**) microarray datasets (GSE74530, GSE30784) and (**b**,**d**) RNA-seq datasets (GSE246050, GSE150469). Rows represent genes, and columns represent individual samples; colors indicate row-scaled log2 expression (red, higher; blue, lower). Sample groups (normal vs. tumor) are indicated by the top annotation bar, and ZNF662 is highlighted in the side annotation.

Volcano plots illustrating these distributions are shown in Figure 1. A Venn diagram (Figure 2a,b) highlights 25 common DEmRNAs (15 up, 10 down) across the four mRNA datasets, which were carried forward as the consensus mRNA pool for downstream miRNA target filtering and ceRNA network construction. Among these, 21 DEmRNAs (Figure 2c) that were also recovered as predicted targets in the miRDB and TargetScan sets were considered high-confidence consensus genes; Figure 3 shows heatmaps of these 21 intersecting DEmRNAs in the microarray and RNA-seq datasets.

### 2.2. Target Prediction and Intersection

MiRDB and TargetScan predicted 13,102 and 17,399 mRNA targets, respectively, for the 56 DEmiRNAs. Taking the intersection of TargetScan and miRDB predictions and then restricting to the 25 consensus DEmRNAs yielded 21 common mRNA targets. For lncRNAs, starBase (ENCORI) yielded 528 lncRNA targets supported by CLIP-seq, and intersecting these with the DElncRNAs identified 22 lncRNAs. Together, these 21 mRNAs and 22 lncRNAs are both differentially expressed and computationally supported as miRNA targets, representing high-confidence candidates for ceRNA network construction and forming the core pool for subsequent survival and functional analyses (Figure 2c,d).

### 2.3. ceRNA Network

**Figure 4:**
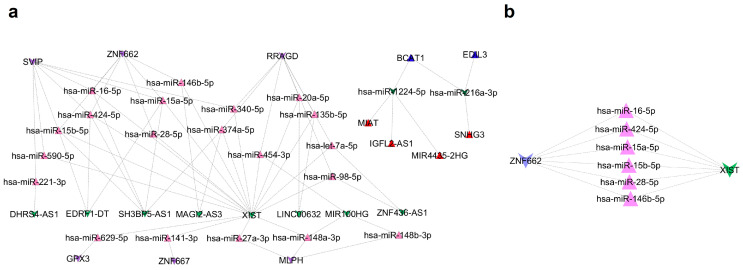
ceRNA network and XIST–miRNA–ZNF662 subnetwork. (**a**) lncRNA–miRNA–mRNA ceRNA network showing the relationships among the eight DEmRNAs, 22 DEmiRNAs, and 12 DElncRNAs. (**b**) ZNF662-centered subnetwork highlighting the core XIST–miRNA–ZNF662 axis. Node types are distinguished as lncRNAs, miRNAs, and mRNAs. Upregulated transcripts in tumors are shown in warm colors (red lncRNAs, pink miRNAs, and blue mRNAs), whereas downregulated transcripts are shown in cool colors (light green lncRNAs, dark green miRNAs, and purple mRNAs). Triangles indicate upregulated nodes, and inverted triangles indicate downregulated nodes. Dashed edges denote predicted lncRNA–miRNA or miRNA–mRNA interactions used to construct the putative ceRNA network.

Applying the ceRNA hypothesis to the consensus DEmRNAs yielded a network comprising 22 DEmiRNAs and 12 DElncRNAs (20 up, 2 down; and 8 up, 4 down, respectively). Upregulated DEmiRNAs targeted six downregulated DEmRNAs, while downregulated DEmiRNAs targeted two upregulated DEmRNAs, yielding eight DEmRNAs in the final lncRNA–miRNA–mRNA ceRNA network (Figure 4). These eight mRNAs, together with their interacting miRNAs and lncRNAs, formed the core ceRNA network for subsequent survival and functional analyses. To improve interpretability, the ceRNA network was visualized with explicit separation of lncRNA, miRNA, and mRNA nodes and with edges denoting predicted lncRNA–miRNA or miRNA–mRNA interactions.

### 2.4. Survival Association

**Figure 5:**
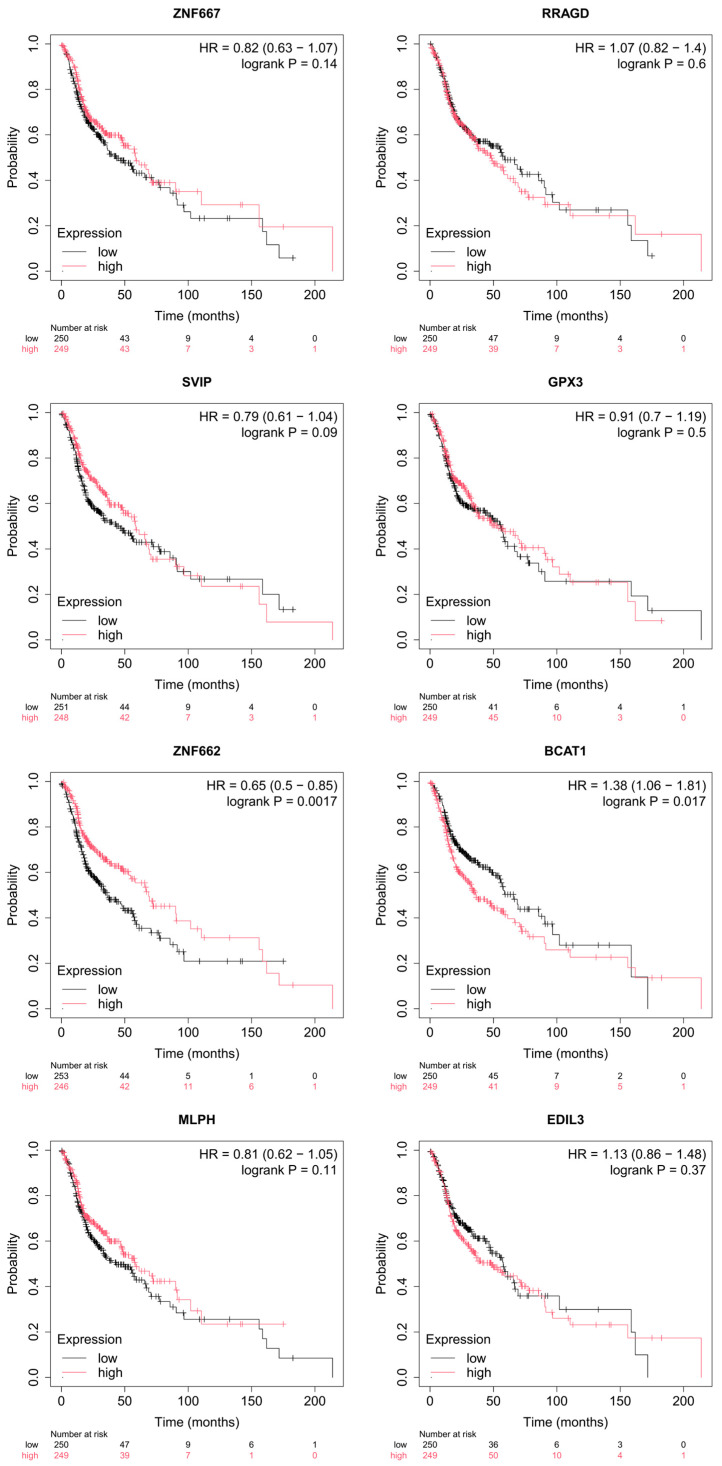
Survival associations for ceRNA network candidate mRNAs in TCGA-HNSC. Kaplan–Meier overall survival curves generated using the Kaplan–Meier Plotter web platform based on TCGA head and neck squamous cell carcinoma (TCGA-HNSC) RNA-seq data (*n* = 499). Patients were stratified into high (red) and low (black) expression groups for each of the eight differentially expressed mRNAs in the core ceRNA network (ZNF662, GPX3, BCAT1, EDIL3, RRAGD, SVIP, ZNF667, and MLPH), using the median expression value as the cut-off. Hazard ratios (HRs), 95% confidence intervals, and log-rank *p*-values are displayed in each panel; *p* < 0.05 was considered statistically significant.

Kaplan–Meier analysis of TCGA head and neck squamous cell carcinoma (TCGA-HNSC) tumors (*n* = 499; Figure 5) highlighted ZNF662 as the most consistent prognostic marker among the eight candidates for mRNAs. High ZNF662 expression was associated with a favorable outcome, showing a significant improvement in overall survival (HR ≈ 0.65, 95% CI ≈ 0.50–0.85, log-rank *p* ≈ 0.0017). In contrast, high BCAT1 expression was linked to poorer overall survival (HR ≈ 1.38, 95% CI ≈ 1.06–1.81, *p* ≈ 0.017), whereas GPX3, EDIL3, RRAGD, SVIP, ZNF667, and MLPH did not show significant associations with overall survival (all *p* > 0.05). The association for ZNF662 was not only protective (HR < 1) but also statistically stronger than that of BCAT1, as reflected by the lower *p*-value, further supporting ZNF662 as the most robust survival-associated candidate in this set. Because ZNF662 also showed consistent downregulation in OSCC across multiple GEO datasets, suggesting a tumor suppressor–like role, we selected it for a detailed follow-up.

### 2.5. Diagnostic Performance and External Validation of ZNF662

**Figure 6:**
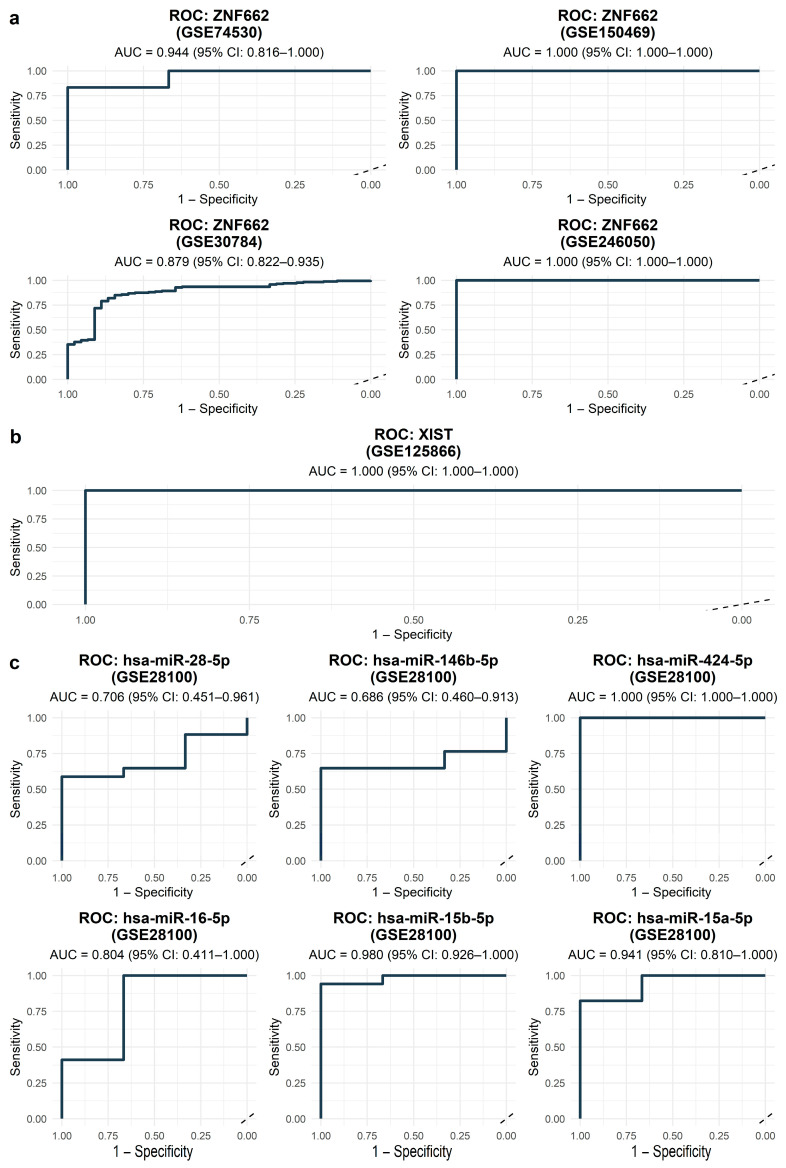
ROC curves for diagnostic performance of XIST–miRNA–ZNF662 axis components. (**a**) ZNF662 distinguishing OSCC from non-tumor samples in four GEO datasets (GSE74530, GSE150469, GSE30784, and GSE246050). (**b**) XIST in GSE125866. AUC values and 95% confidence intervals are shown in each panel. (**c**) Axis miRNAs (hsa-miR-15b-5p, hsa-miR-15a-5p, hsa-miR-28-5p, hsa-miR-146b-5p, hsa-miR-424-5p, and hsa-miR-16-5p) in GSE28100.

Receiver operating characteristic (ROC) analysis across the GEO datasets analyzed in this study supported diagnostic potential for components of the XIST–miRNA–ZNF662 axis (Figure 6). ZNF662 discriminated OSCC from non-tumor tissue with AUC = 0.944 (95% CI, 0.816–1.00) in GSE74530, AUC = 1.00 (95% CI, 1.00–1.00) in GSE150469, and AUC = 0.879 (95% CI, 0.822–0.935) in the larger GSE30784 cohort (Figure 6a). Axis miRNAs also showed diagnostic signals in GSE28100 (Figure 6c): hsa-miR-15b-5p (AUC = 0.98, 95% CI, 0.926–1.00), hsa-miR-15a-5p (AUC = 0.941, 95% CI, 0.81–1.00), and hsa-miR-424-5p (AUC = 1.00, 95% CI, 1.00–1.00) achieved excellent discrimination; hsa-miR-16-5p showed intermediate performance (AUC = 0.804, 95% CI, 0.411–1.00); whereas hsa-miR-28-5p (AUC = 0.706, 95% CI, 0.451–0.961) and hsa-miR-146b-5p (AUC = 0.686, 95% CI, 0.46–0.913) exhibited more modest but still suggestive diagnostic performance. Consistent with its central position in the network, XIST also demonstrated perfect diagnostic separation (AUC = 1.00, 95% CI, 1.00–1.00; Figure 6b).

**Figure 7:**
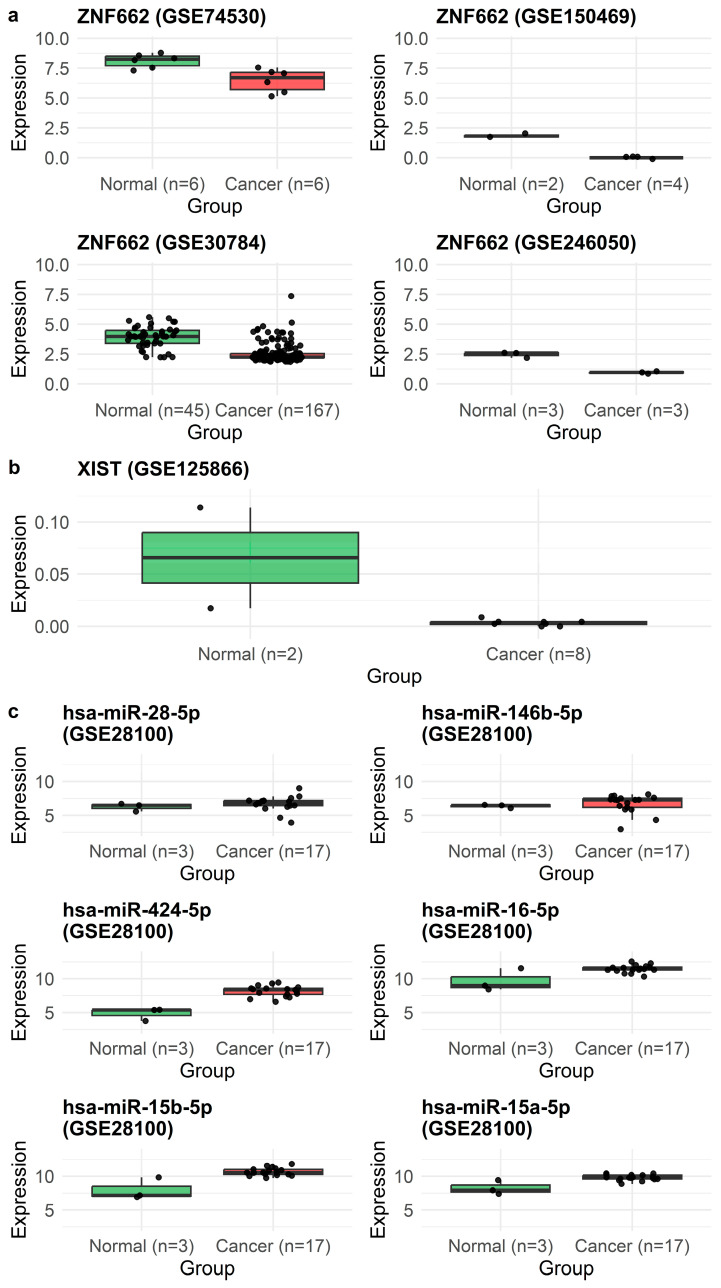
Expression of ZNF662, axis miRNAs, and XIST in OSCC versus normal samples. Boxplots show log2-transformed expression values for tumor (red) and normal (green) samples in GEO datasets with significant tumor–normal differences (*p* < 0.05). (**a**) ZNF662 expression in GSE74530 (*n* = 6 normal, 6 OSCC), GSE150469 (*n* = 2 immortalized oral keratinocyte lines, 5 OSCC cell lines), GSE30784 (*n* = 45 normal, 167 OSCC), and GSE246050 (*n* = 3 adjacent normal, 3 OSCC). (**b**) XIST expression in GSE125866 (*n* = 2 normal, 8 OSCC). (**c**) Expression of axis miRNAs hsa-miR-28-5p, hsa-miR-146b-5p, hsa-miR-424-5p, hsa-miR-16-5p, hsa-miR-15a-5p, and hsa-miR-15b-5p in GSE28100 (*n* = 3 normal, 17 OSCC). Boxes indicate the median and interquartile range (IQR); whiskers extend to 1.5 × IQR, and points represent individual samples.

**Figure 8:**
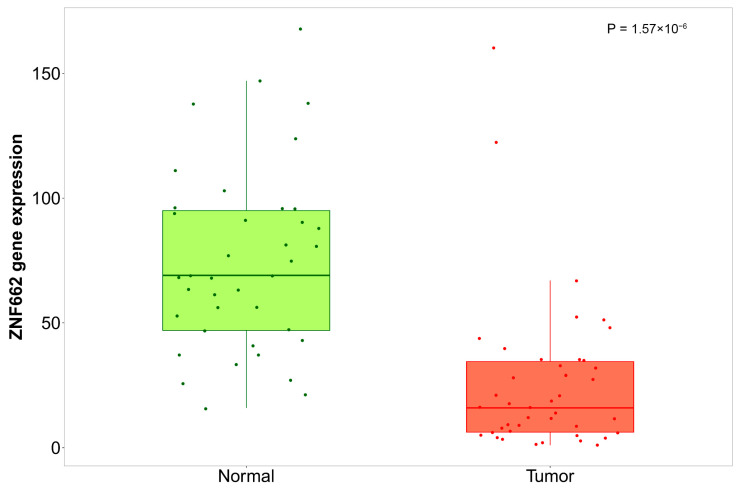
External RNA-seq validation of ZNF662 expression in head and neck cancer. Boxplots show log2-transformed ZNF662 expression in normal versus tumor head and neck tissues obtained from RNA-seq data using the TNMplot.com web tool. Tumor samples are shown in red and normal samples in green. Boxes indicate the median and interquartile range (IQR), whiskers extend to 1.5 × IQR, and points represent individual samples. Differences between normal and tumor tissues confirm the downregulation of ZNF662 observed in GEO microarray datasets.

In line with these ROC metrics, independent expression profiling showed that ZNF662 was significantly downregulated in OSCC compared with normal mucosa across GSE74530, GSE150469, GSE246050, and GSE30784 (Figure 7a). Expression patterns for all six axes of miRNAs in GSE28100 are shown in Figure 7c, with several (hsa-miR-146b-5p, hsa-miR-424-5p, hsa-miR-15a-5p, and hsa-miR-15b-5p) upregulated in OSCC, and XIST expression readily detectable in the GSE125866 lncRNA dataset (Figure 7b). External validation using TNMplot.com confirmed that ZNF662 is significantly downregulated in tumors compared with normal tissues, further supporting its role as a survival-associated tumor suppressor candidate in OSCC (Figure 8).

### 2.6. Functional Enrichment Findings

**Figure 9:**
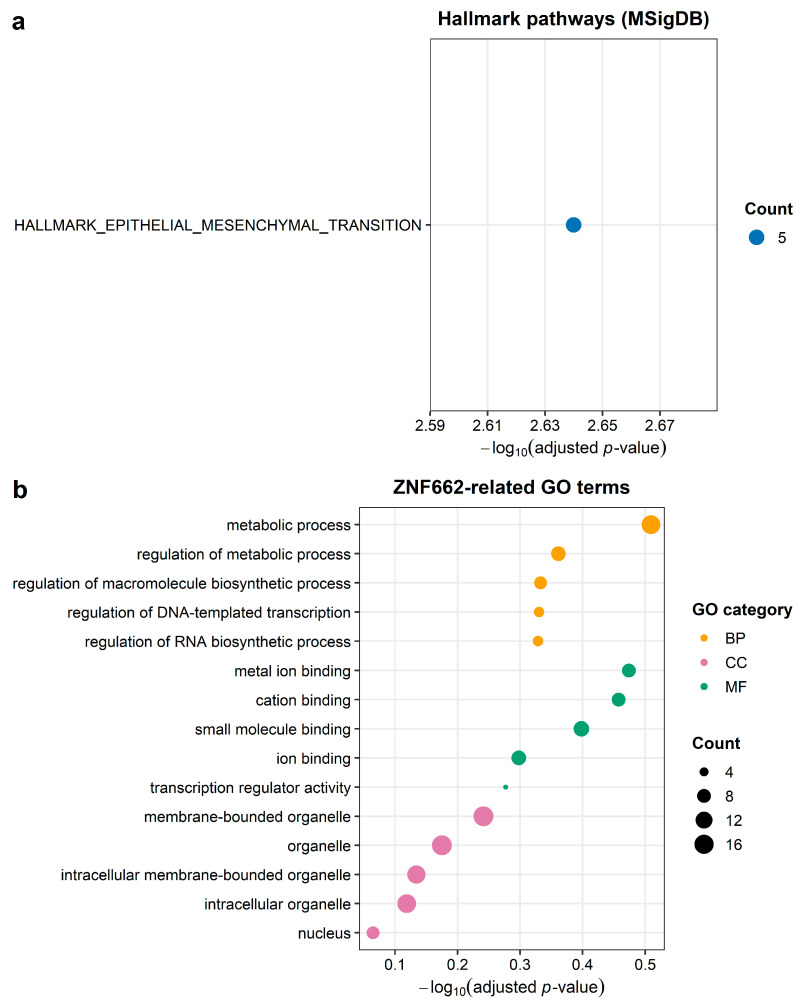
Dot plots of functional enrichment analyses. (**a**) Hallmark pathway over-representation analysis (MSigDB H collection) for the 21-gene panel. The *x*-axis shows the number of genes from the panel mapped to each Hallmark term, and bubble size also reflects gene count. Bubble fill indicates −log10 (adjusted *p*-value), with darker blue representing stronger enrichment. (**b**) Gene Ontology (GO) enrichment for the same 21-gene panel. The bubble plot shows the top enriched biological process (BP, green), molecular function (MF, orange), and cellular component (CC, purple) terms. The *x*-axis denotes gene count, bubble size scales with gene count, and each horizontal facet corresponds to one GO category.

At the network level, Hallmark Epithelial– Mesenchymal Transition (Hallmark EMT) was the only significantly enriched program (Figure 9a), consistent with the established role of epithelial–mesenchymal transition in tumor invasion and metastasis. To place the core XIST–miRNA–ZNF662 axis in a broader biological context, we performed enrichment analysis on the network-level 21-gene panel derived from the filtered ceRNA network. This panel was used only for the exploratory pathway analysis and does not imply that all included genes are direct effectors of ZNF662. Gene Ontology terms associated with genes correlated with ZNF662 suggested possible involvement in metabolic and transcriptional regulation, but no pathways remained significant after multiple testing corrections, so these patterns should be regarded as exploratory (Figure 9b).

## 3. Discussion

Our integrative analysis of GEO differential expression and TCGA survival data highlights an XIST–miRNA–ZNF662 competing endogenous RNA (ceRNA) axis with both diagnostic and prognostic potential in oral squamous cell carcinoma (OSCC). By combining lncRNA, miRNA, and mRNA expression across multiple GEO datasets with three complementary target-prediction resources and TCGA-HNSC survival information, we reduced a large set of differentially expressed RNAs to a compact ceRNA network in which an XIST-centered subnetwork converges on ZNF662. Network-level interrogation using the Hallmark collection from the Molecular Signatures Database (MSigDB) identified epithelial–mesenchymal transition (EMT) as the only significantly enriched program, indicating that genes in this ceRNA network collectively map to a core EMT signature. Given the established role of EMT as a key enabler of invasion and metastasis in OSCC and other carcinomas, governed by pathways such as TGFβ, Wnt/β-catenin, Notch, and receptor tyrosine kinase signaling [20,21], this convergence suggests that dysregulated ceRNA interactions may contribute to EMT-linked tumor aggressiveness.

Importantly, the network-level gene set used for functional enrichment was included to provide broader biological context for the filtered ceRNA network and should not be interpreted as a set of direct downstream targets of ZNF662. Compared with previously reported XIST-related ceRNA networks in OSCC, our study differs by integrating multiple independent GEO datasets across lncRNA, miRNA, and mRNA layers; intersecting three target-prediction resources; and jointly evaluating tumor–normal expression, diagnostic performance, and survival associations.

Within the mRNA layer, ZNF662 emerged as the most consistent prognostic signal. ZNF662 was downregulated in tumors, and higher expression was associated with improved overall survival in TCGA-HNSC, compatible with a tumor-suppressive role in OSCC. Although gene-centric enrichment around ZNF662-associated genes did not yield statistically significant pathways after multiple-testing correction, TCGA-HNSC expression analyses revealed reduced tumor expression; external validation using TNM-plot.com confirmed significantly lower ZNF662 levels in tumors compared with normal head and neck tissues; and independent studies report promoter hypermethylation–mediated silencing and tumor-suppressive activity in other tumor types [22,23,24,25]. Together, these data nominate ZNF662 as a robust survival-associated marker whose biology warrants further mechanistic dissection in OSCC and, to our knowledge, provide the first systematic evidence linking ZNF662 expression to prognosis in head and neck cancer. However, these findings indicate an association with overall survival in TCGA-HNSC and do not establish that ZNF662 or the proposed XIST–miRNA–ZNF662 axis is an independent prognostic factor.

The ceRNA network also implicates six miRNAs—hsa-miR-28-5p, hsa-miR-146b-5p, hsa-miR-424-5p, hsa-miR-16-5p, hsa-miR-15b-5p, and hsa-miR-15a-5p—as potential regulators of ZNF662 (and partners of XIST) via shared microRNA response elements. Published work supports a mixed oncogenic and tumor-suppressive landscape for these miRNAs, with context-dependent effects on proliferation, apoptosis, EMT and angiogenesis: hsa-miR-28-5p can promote proliferation and EMT in ovarian cancer, yet restrain growth in colorectal and nasopharyngeal models [26,27,28]; hsa-miR-146b-5p promotes EMT, and invasion by repressing SMAD4, and engaging TGFβ signaling in thyroid and esophageal cancers [29,30,31]; the hsa-miR-15/16 family are canonical tumor suppressors, inducing apoptosis and cell-cycle arrest via targets such as BCL2, MCL1, CCND1, and WNT3A [32,33,34]; and hsa-miR-424-5p participates in cell-cycle control and angiogenesis via the HIF–VEGF axis, again with context-dependent roles [35,36]. In our data, several axis miRNAs were upregulated in OSCC and showed strong diagnostic performance, consistent with a model in which increased miRNA expression contributes to repression of ZNF662 and EMT-related biology within this ceRNA module.

LncRNA XIST was downregulated in our datasets and—together with ZNF662 and several of the six miRNAs—showed high diagnostic accuracy, with AUCs typically above 0.8 for distinguishing OSCC from non-tumor samples in the GEO series analyzed. The diagnostic potential of this axis is supported not only by the performance of individual transcripts but also by their coordinated tumor-versus-normal dysregulation within a biologically connected ceRNA module. In other words, XIST, ZNF662, and the shared miRNAs were prioritized because they combine consistent differential expression, predicted regulatory connectivity, and reproducible discriminatory performance across independent datasets, making the axis more informative than isolated single-marker candidates. Prior OSCC work demonstrates that XIST can function as a ceRNA, for example, by sponging hsa-miR-455-3p to restore BTG2 and restrain tumor growth, and clinical data indicate that salivary XIST is detectable and correlates with OSCC risk in a sex-specific manner, underscoring its potential non-invasive biomarker utility. Conversely, context-dependent upregulation of XIST has also been reported in other cohorts and tumor types, highlighting platform-, population-, and disease-specific variability [37,38,39,40]. Our findings; therefore, add to a complex picture in which XIST may have tumor-suppressive or oncogenic roles depending on cellular context, interacting miRNAs, and downstream targets such as ZNF662 and extend this by placing XIST and ZNF662 within a single, EMT-linked ceRNA axis in OSCC.

Beyond the specific XIST–miRNA–ZNF662 module, our study has several distinguishing features. First, rather than relying on a single cohort, we integrated one miRNA, one lncRNA, and four mRNA GEO datasets and required concordant differential expression across mRNA series, thereby reducing dataset-specific noise and platform bias. Second, we constructed the ceRNA network by intersecting TargetScan-, miRDB-, and starBase-derived interactions with differentially expressed genes, yielding a compact, higher-confidence set of lncRNA–miRNA–mRNA triplets instead of a large, weakly supported network. Third, we jointly evaluated tumor–normal expression, diagnostic performance, and TCGA-HNSC survival associations, enabling prioritization of axes with both diagnostic and prognostic relevance rather than expression changes alone. This integrated prioritization strategy also provides the rationale for the proposed diagnostic utility of the XIST–miRNA–ZNF662 axis because it identifies a coordinated expression pattern linked to disease status across multiple datasets rather than a single dysregulated transcript. Fourth, functional interrogation using curated gene sets identified biologically meaningful programs linked to tumor progression, providing a network-level context for the proposed ceRNA interactions. Finally, this integrated workflow converged on an XIST–miRNA–ZNF662 axis that, to our knowledge, has not previously been described in OSCC, highlighting ZNF662 as a putative tumor suppressor with favorable survival associations and strong diagnostic performance within an EMT-enriched ceRNA network.

### Clinical Relevance and Limitations

Our findings suggest that coordinated dysregulation of XIST, the six axis miRNAs, and ZNF662 may be informative both for distinguishing OSCC from non-tumor tissue and for stratifying patient prognosis, with potential application as part of a multimarker panel rather than as standalone biomarkers. XIST and ZNF662 showed high diagnostic accuracy, while higher ZNF662 expression was consistently associated with improved survival in TCGA-HNSC. However, because these survival analyses were based on Kaplan–Meier estimates without multivariable adjustment, the present study does not establish that ZNF662 or the proposed XIST–miRNA–ZNF662 axis is an independent prognostic factor. Because survival analyses were performed in the broader TCGA-HNSC cohort rather than an OSCC-only cohort, these prognostic findings should be interpreted as supportive rather than definitive evidence for OSCC-specific outcome associations. From a clinical perspective, existing evidence that XIST is detectable in saliva and associated with OSCC risk points to a plausible route for non-invasive testing in oral healthcare settings [37,38,39,40]. However, in the present work we analyzed only publicly available tissue-based GEO and TCGA datasets and did not recruit or profile an independent patient cohort.

This study is exploratory and based entirely on retrospective, de-identified transcriptomic data. The included cohorts differ in platform, sample processing, and clinical annotation; key covariates such as treatment, human papillomavirus status, and comorbidities were not uniformly available, precluding formal adjustment for these factors. All interactions within the proposed axis are supported by integrated expression analysis, in silico target prediction, and correlation patterns only and were not validated by qRT-PCR, luciferase reporter assays, RNA immunoprecipitation, or gain- and loss-of-function experiments in OSCC models. As such, our results should be regarded as hypothesis-generating. Future studies should validate this axis in independent OSCC cohorts with matched clinicopathological data and standardized tissue and saliva sampling and should test the functional relationship among XIST, the candidate miRNAs, and ZNF662 using molecular perturbation and reporter-based assays.

## 4. Materials and Methods

### 4.1. Data Compilation

Expression profiles from one lncRNA dataset, one miRNA dataset, and four mRNA datasets were retrieved from the Gene Expression Omnibus (GEO) [41,42]. Only samples from oral cancer and normal tissue or cell lines were included; samples of leukoplakia, oral submucous fibrosis, and other pre-malignant conditions were excluded. Because datasets originated from distinct platforms, differential expression analyses were conducted within each dataset without cross-dataset batch correction. Dataset accession, platform, and sample composition are given in Table 2.

### 4.2. Differential Expression Analysis

Within each dataset, tumor versus normal comparisons were performed using linear modeling with empirical Bayes moderation as implemented in the limma package in R, accessed via the GEO2R interface for microarray series and replicated in local R sessions for RNA-seq series [43,44]. For microarray datasets (GSE30784, GSE74530), GEO2R uses limma to (i) apply log2 transformation where required, (ii) perform quantile normalization of expression values, and (iii) fit probe-wise linear models with empirical Bayes shrinkage of variances. For RNA-seq datasets (GSE150469, GSE246050, GSE125866), we analyzed GEO-provided count or expression matrices with limma package (version 3.64.0; Bioconductor release 3.21) using the voom transformation when raw counts were available, followed by the same linear modeling and empirical Bayes pipeline. In all datasets, design matrices encoded tumor versus normal status, and contrasts were defined to estimate differential expression for tumors relative to normal samples. Differentially expressed genes were identified using |log_2_FC| > 1 and false discovery rate (FDR) < 0.05 as cut-offs. Visualizations of the results were carried out using the EnhancedVolcano, ggplot2, and pheatmap (v1.0.12) packages in R [45,46,47]. Intersecting DEmRNAs across the two microarrays and two RNA-seq mRNA datasets yielded a consensus set of 25 common DEmRNAs (15 upregulated, 10 downregulated). Of these, 21 DEmRNAs that also overlapped with the miRNA target predictions (detailed in the next subsection) were retained as high-confidence consensus genes. A heatmap of these 21 intersecting DEmRNAs illustrates tumor–normal clustering patterns. 

### 4.3. Prediction of miRNA–mRNA and lncRNA–miRNA Interactions

For miRNA–mRNA interaction prediction, we used TargetScanHuman Release 8.0 (TargetScan 8.0) (https://www.targetscan.org/vert_80/ (accessed on 1 July 2025)) and miRDB Version 6.0 (https://mirdb.org/index.html (accessed on 1 July 2025)), including all predicted miRNAs without applying additional score-based filtering. TargetScan and miRDB use complementary algorithms (conservation-based vs. machine learning-based), so intersecting their predictions provides higher-confidence miRNA–mRNA targets than either tool alone [48,49]. For lncRNA–miRNA interactions, we used the ENCORI (Encyclopedia of RNA Interactomes) (starBase v3.0) platform (https://rnasysu.com/encori/ (accessed on 5 July 2025 )) and retained only pairs supported by crosslinking immunoprecipitation sequencing (CLIP-seq) evidence [50]. Predicted targets were then intersected with the consensus differentially expressed (DE) genes to define candidate lncRNA–miRNA–mRNA triplets, as intersecting predicted targets with DE genes reduces false-positive in silico predictions and enriches for biologically relevant interactions in OSCC.

### 4.4. ceRNA Network Construction

Following the ceRNA hypothesis, we identified directionally consistent triplets in which lncRNAs, and mRNAs changed in the same direction and opposite to their shared miRNAs. Specifically, downregulated lncRNAs were paired with upregulated DEmiRNAs and their corresponding downregulated DEmRNAs, and vice versa. All interactions meeting these criteria were retained, allowing many-to-many relationships between lncRNAs, miRNAs, and mRNAs. The resulting lncRNA–miRNA–mRNA interaction network was visualized in Cytoscape (v3.9.1) [51].

### 4.5. Survival Analyses

Associations between RNA expression and patient prognosis were evaluated using the Kaplan–Meier Plotter web platform (https://kmplot.com/analysis (accessed on 10 July 2025 )), which provides standardized survival analyses based on TCGA and other public datasets [52]. For head and neck squamous cell carcinoma, we used the RNA-seq cohort and the platform’s default log2-transformed expression values. Analyses included all tumors with available RNA-seq expression and survival data, without restriction to anatomical subsite. Overall survival (OS) was assessed using Kaplan–Meier survival curves. Hazard ratios (HRs) with 95% confidence intervals (CIs) and log-rank *p*-values were reported, with statistical significance defined as *p* < 0.05. When multiple candidate genes were screened, analyses were considered exploratory, and interpretation emphasized effect sizes, with ZNF662 prioritized for detailed evaluation. Survival analyses were performed using the TCGA-HNSC cohort because sufficiently powered OSCC-specific datasets with matched transcriptomic and survival data were not available for the present study.

### 4.6. Diagnostic Evaluation and External Validation of ZNF662

Diagnostic performance of XIST, ZNF662, and the six-axis miRNAs was evaluated in R using the pROC (version 1.19.0.1), dplyr (version 1.1.4), and ggplot2 packages (version 4.0.1) [45,53]. All ROC analyses used log2-transformed expression values, consistent with the differential expression pipelines. Receiver operating characteristic (ROC) curves were generated for each marker to distinguish oral squamous cell carcinoma (OSCC) from normal samples in the relevant GEO datasets: for ZNF662, GSE30784 (167 OSCC, 45 normal); GSE74530 (6 OSCC, 6 matched normal), GSE150469 (5 OSCC cell lines, 2 immortalized oral keratinocytes); and GSE246050 (3 OSCC, 3 adjacent normal); for the six axis miRNAs, GSE28100 (17 OSCC, 3 normal); and for XIST, GSE125866 (8 OSCC, 2 normal). The area under the curve (AUC) was calculated with automatic orientation to ensure that higher expression corresponded to the cancer group, and an AUC greater than 0.8 was considered to indicate strong discriminatory ability. Ninety-five percent confidence intervals (95% CIs) for AUC values were obtained using pROC’s bootstrap procedure (version 1.19.0.1) (typically 400 stratified resamples, adaptively reduced for very small class sizes) or DeLong’s method where sample sizes permitted. Model robustness was assessed using stratified K-fold cross-validation (K = 2–5; up to 10 repeats), aggregating out-of-fold predictions to obtain cross-validated ROC curves and 95% CIs. Expression differences between tumor and normal samples were visualized using ggplot2 boxplots for each marker in the same GEO series (ZNF662 in GSE30784, GSE74530, GSE150469, and GSE246050; axis miRNAs in GSE28100; XIST in GSE125866). Validation of ZNF662 expression differences between normal and tumor tissues was performed using the TNMplot.com web tool (https://www.tnmplot.com (accessed on 20 July 2025)) [54]. The RNA-seq module was used to compare ZNF662 expression between normal and head and neck squamous cell carcinoma samples, and expression distributions were visualized as box plots. Statistical significance of normal–tumor differences was assessed using the default test implemented in TNMplot.com, with *p* < 0.05 considered significant.

### 4.7. Functional Enrichment Analysis

Functional enrichment was performed using the clusterProfiler (version 4.18.2) and enrichR (version3.4) packages. Molecular Signatures Database (MSigDB) Hallmark gene sets, including the epithelial–mesenchymal transition (Hallmark EMT) program, were queried to identify network-level biological programs. For network-level enrichment, we analyzed the pro-tein-coding gene set retained after ceRNA-network filtering (hereafter referred to as the 21-gene panel). This panel was used to provide broader biological context for the filtered ceRNA network and should not be interpreted as a set of direct downstream targets of ZNF662. Gene Ontology (GO) and Kyoto Encyclopedia of Genes and Genomes (KEGG) pathways were analyzed for complementary functional context [55,56,57]. Over-representation analyses for both the ceRNA-network genes and the ZNF662-correlated genes used the union of all DEmRNAs identified across the four expression datasets as the background gene set significance was defined as FDR q < 0.05.

### 4.8. Statistics and Reproducibility

All tests were two-sided unless otherwise specified. We report that *n*, effect sizes, and 95% CIs where applicable. Multiple testing was controlled via Benjamini–Hochberg FDR for differential expression, enrichment, and any multi-feature screening (e.g., testing multiple RNAs in survival/ROC). GEO microarray series (GSE30784, GSE74530) were analyzed with limma, using log2 transformation (where required), quantile normalization, and linear models with empirical Bayes variance moderation; RNA-seq series (GSE150469, GSE246050, GSE125866) were analyzed from GEO-provided matrices with limma, applying voom to raw counts wherever available and then fitting the same limma linear modeling framework. All analyses were performed in R (version 4.5.0) (R Core Team, 2023; Vienna, Austria) within RStudio Desktop (Posit, Boston, MA, USA); specific package versions are provided in the references [43,44,45,46,47,53,54,55,56,57].

## 5. Conclusions

By integrating multiple GEO lncRNA, miRNA, and mRNA datasets with TCGA-HNSC clinical data, we constructed an OSCC-focused ceRNA network and identified an XIST–miRNA–ZNF662 axis with both diagnostic and prognostic relevance. XIST, several associated miRNAs, and the mRNA ZNF662 showed coordinated dysregulation between tumor and non-tumor samples and strong diagnostic performance for distinguishing OSCC, while higher ZNF662 expression was favorably associated with overall survival. Network-level analysis using curated gene sets indicated that genes within this axis are linked to biological processes relevant to tumor progression, and external validation using TNMplot.com confirmed significantly lower ZNF662 expression in tumors compared with normal tissues.

These findings nominate XIST and its associated miRNAs, converging on the putative tumor suppressor ZNF662, as components of a ceRNA-based biomarker panel that merits further investigation in OSCC. However, this work is based entirely on retrospective analyses of publicly available, de-identified datasets and does not include an independent patient cohort or experimental validation; it should therefore be regarded as hypothesis-generating. Prospective studies with standardized tissue and saliva sampling, comprehensive clinical annotation, and functional perturbation of XIST, the axis miRNAs, and ZNF662 will be essential to determine whether this ceRNA axis can be developed into robust, clinically useful biomarkers or therapeutic targets in oral cancer care.

## Figures and Tables

**Table 1:** Summary of differentially expressed RNAs (DE RNAs) across GEO datasets.

Dataset	RNA Type	Platform	Total DERNAs	Upregulated	Downregulated
GSE28100	miRNA	Microarray	56 DEmiRNAs	45	11
GSE125866	lncRNA	RNA-seq	2910 DElncRNAs	1480	1430
GSE150469	mRNA	RNA-seq	680 DEmRNAs	267	413
GSE246050	mRNA	RNA-seq	3905 DEmRNAs	1617	2288
GSE74530	mRNA	Microarray	3412 DEmRNAs	2439	973
GSE30784	mRNA	Microarray	3866 DEmRNAs	1993	1873

DE, differentially expressed; DEmiRNA, differentially expressed miRNA; DElncRNA, differentially expressed lncRNA; DEmRNA, differentially expressed mRNA. Differential expression threshold: |log_2_FC| > 1 and FDR < 0.05.

**Table 2:** GEO datasets included in the study.

Dataset ID	RNA Type	Platform	Sample Composition
GSE30784	mRNA (microarray)	Affymetrix HG-U133 Plus 2.0 (GPL570)	167 OSCC, 45 normal
GSE74530	mRNA (microarray)	Affymetrix HG-U133 Plus 2.0 (GPL570)	6 OSCC, 6 matched normal (paired)
GSE150469	mRNA (RNASeq)	Illumina Genome Analyzer II (GPL9115)	5 OSCC lines vs. 2 immortalized oral keratinocytes
GSE246050	mRNA (RNASeq)	HiSeq X Ten	3 OSCC, 3 adjacent normal
GSE28100	miRNA (microarray)	Agilent-021827 Human miRNA V3	17 OSCC, 3 normal
GSE125866	lncRNA (RNASeq)	Illumina HiSeq 4000	8 OSCC, 2 normal

OSCC, oral squamous cell carcinoma; RNASeq, RNA sequencing.

## Data Availability

The datasets analyzed in this study are publicly available. GEO accessions: GSE30784, GSE74530, GSE150469, GSE246050, GSE28100 and GSE125866 (https://www.ncbi.nlm.nih.gov/geo/ (accessed on 23 June 2025). All analysis scripts and configuration files used to generate the results in this manuscript are publicly available at: https://github.com/nowsheenb2 (accessed on 1 September 2025).

## Abbreviations

AUC	Area under the curve
ceRNA	Competing endogenous RNA
CI	Confidence interval
CLIP	Crosslinking immunoprecipitation
DE	Differentially expressed
ENCORI	Encyclopedia of RNA Interactomes
EMT	Epithelial–mesenchymal transition
FDR	False discovery rate
GEO	Gene Expression Omnibus
GO	Gene Ontology
HNSC	Head and neck squamous cell carcinoma
HR	Hazard ratio
KEGG	Kyoto Encyclopedia of Genes and Genomes
lncRNA	Long noncoding RNA
miRNA	MicroRNA
MRE	MicroRNA response element
MSigDB	Molecular Signatures Database
OSCC	Oral squamous cell carcinoma
ROC	Receiver operating characteristic
TCGA	The Cancer Genome Atlas
TPM	Transcripts per million
